# Solvent-Free Synthesis of Magnetic Sewage Sludge-Derived Biochar for Heavy Metal Removal from Wastewater

**DOI:** 10.3390/ijerph20010155

**Published:** 2022-12-22

**Authors:** Jiayi Tian, Kexin Guo, Yucan Sun, Ruoxi Lin, Tan Chen, Bing Zhang, Yifei Liu, Ting Yang

**Affiliations:** 1 College of Life and Environmental Sciences, Minzu University of China, Beijing 100081, China; 2 Technical Centre for Soil, Agriculture and Rural Ecology and Environment, Ministry of Ecology and Environment, Beijing 100012, China

**Keywords:** magnetic biochar, toxic metals, adsorption performance, kinetics, isotherms, mechanisms

## Abstract

The commonly used two-step and one-pot synthesis methods for producing biochar require the use of iron salt solutions, resulting in the undesirable consequences of energy consumption for dewatering and potential pollution risks. To address this drawback, a magnetic sewage sludge-derived biochar (MSBC-2) was synthesized by a solvent-free method in this study. The pseudo-second-order kinetic model and Langmuir model provided the best fit to the experimental data, implying a monolayered chemisorption process of Pb^2+^, Cd^2+^and Cu^2+^ onto MSBC-2. As the reaction temperature increased from 25 °C to 45 °C, the maximum adsorption capacities increased from 113.64 mg·g^−1^ to 151.52 mg·g^−1^ for Pb^2+^, from 101.01 mg·g^−1^ to 109.89 mg·g^−1^ for Cd^2+^ and from 57.80 mg·g^−1^ to 74.07 mg·g^−1^ for Cu^2+^, respectively. Thermodynamic parameters (Δ*G*^0^ < 0, Δ*S*^0^ > 0, Δ*H*^0^ > 0) revealed that the adsorption processes of all three metals by MSBC-2 were favourable, spontaneous and endothermic. Surface complexation, cation-π interaction, ion exchange and electrostatic attraction mechanisms were involved in the adsorption of Pb^2+^, Cd^2+^ and Cu^2+^ onto MSBC-2. Overall, this study will provide a new perspective for the synthesis of magnetic biochar and MSBC-2 shows great potential as an adsorbent for heavy metal removal.

## 1. Introduction

Industrial wastewater often contains various heavy metals, including Pb^2+^, Cd^2+^ and Cu^2+^, etc. Heavy metals are not biodegradable and can be enriched in humans through the food chain, leading to a serious impact on the aquatic environment and public health [1,2]. Current heavy metal removal approaches include ion exchange, co-precipitation, membrane filtration and adsorption, etc. [3,4,5,6,7]. Among them, adsorption is regarded as one of the most simple, cost-effective and efficient techniques, and has attracted increasing attention. As adsorbents are essential for adsorption applications, various adsorbents have been developed, such as activated carbon, biochar, metal oxides in nanoscale, natural minerals, and polymers [8,9]. Generally, biochar is a carbon-based adsorbent which is generated by the pyrolysis of biomass under anaerobic conditions or in the presence of limited oxygen [10]. Due to its large specific surface area, abundant oxygen-containing functional groups and low cost, biochar has been considered as a promising adsorbent [11,12].

Sewage sludge is a by-product of wastewater treatment plants, which contains various organic pollutants and pathogens [13]. Traditional treatment methods for sewage sludge (e.g., incineration and sanitary landfill) often have high energy consumption, low efficiency and a tendency to cause secondary pollution, thus it is imperative to develop environmentally friendly sludge treatment approaches [14,15]. Lately, sewage sludge has been converted into biochar by pyrolysis and applied to remove heavy metals from wastewater as the adsorbent or to mitigate greenhouse gas emissions as the soil amendment, which has received increasing interest [16]. Previous studies reported that sewage sludge-derived biochar (SBC) was employed as an efficient adsorbent for the removal of Pb, Zn, Cd and Cu from aqueous solution [17,18]. Compared to other biochar, SBC is demonstrated to have more oxygen-containing functional groups (e.g., carboxyl, hydroxyl, and carbonyl groups) and larger specific surface area, because of the large number of microorganisms and organic matters in the sludge [19]. However, it is difficult to separate SBC adsorbent from the aqueous solution after adsorption due to the tiny particle size, restricting its practical application in wastewater treatment.

Despite the difficulty in separating from water, raw biochar has other disadvantages such as small particle size and low density. To eliminate the above shortcomings and enhance the metal removal efficiency, various modification methods (e.g., surface oxidation, impregnation of metal oxides and functionalization) have been used to modify biochar [20,21,22]. Among them, the magnetic modification of biochar offers the potential for rapid separation and recovery in the presence of an external magnetic field. For instance, previous studies have reported that FeCl_3_-modified biochar had excellent magnetic sensitivity and could be separated from the aqueous solution rapidly [23]. In addition, magnetic modification can enhance the metal adsorption performance [19,24,25]. Currently, two-step and one-pot synthesis methods are the most used to produce magnetic biochar [26,27]. For example, the former study successfully used a two-step method to prepare a magnetic tea-based biochar with an iron-containing solution [27]. In terms of the one-pot method, the substrate is usually placed in a magnetic solution containing metal ions for impregnation loading and then pyrolysis is conducted to obtain biochar [28]. However, both require the use of iron salt solutions as post- and pre-treatment reagents, respectively. This caused the inevitable consequences of energy consumption for dewatering, potential pollution risks and operational burden [29,30]. To address this drawback, the development of solvent-free synthesis methods for magnetic biochar gradually gains the attention of researchers.

In this study, a magnetic sludge-derived biochar (MSBC-2) was synthesized based on a solvent-free method. The as-prepared material was characterized by scanning electron microscopy (SEM), Fourier transform infrared spectrometer (FTIR), X-ray photoelectron spectroscopy (XPS), vibrating sample magnetometer (VSM) and Raman spectroscopy. The Pb^2+^, Cd^2+^ and Cu^2+^ were selected as representative heavy metal ions and the effects of pH, temperature, background ionic strength and adsorbent dosage on the metal removal efficiency of MSBC-2 were determined. Combining adsorption kinetics, isotherms, thermodynamics analysis and further characterization results, the adsorption mechanisms of three heavy metals onto MSBC-2 were systematically revealed. This work will provide a new perspective for the synthesis of magnetic biochar with excellent metal adsorption capacities.

## 2. Materials and Methods

### 2.1. Materials

Cadmium (II) nitrate tetrahydrate (Cd(NO_3_)_2_·4H_2_O, >99.0% purity), copper (II) nitrate trihydrate (Cu(NO_3_)_2_·3H_2_O, >99.0% purity) and Fe_3_O_4_ nanoparticles (99.5% purity) were purchased from Shanghai Macklin Biochemical Technology Co., Ltd. (Shanghai, China). Lead (II) nitrate (Pb(NO_3_)_2_, >99.0% purity), sodium nitrate (NaNO_3_, >99.0% purity) and nitric acid (65–68% purity) were purchased from Sinopharm Chemical Reagent Co., Ltd. (Shanghai, China). Ultra-pure water was prepared by the Milli-Q water purification system.

### 2.2. Preparation of Magnetic Sewage Sludge-Derived Biochar

The sewage sludge used in this study was obtained from the No.2 wastewater treatment plant of Kunming City, with a moisture content of 80.20 ± 0.07%, volatile solid of 43.99 ± 0.05% (dry basis) and C/N of 6.48. The moisture content of the sewage sludge decreased to approximately 30% after several days of drying. Sewage sludge was further air-dried to reduce moisture content (<5%), and ground and passed through a 60-mesh sieve.

Subsequently, 5 g of air-dried sewage sludge was mixed with a certain mass of Fe_3_O_4_ nanoparticles and mechanically ground to make a uniform mixture. The mixture was placed in a quartz boat and pyrolyzed at 800 °C for 2 h with a heating rate of 5 °C/min under a N_2_ atmosphere in a tube furnace (SK-G06123K, Tianjin Zhonghuan Furnace Corp, China). The obtained material was cooled to room temperature, then ground and passed through the 60-mesh sieve. In our previous study, the Fe_3_O_4_ nanoparticle content (based on Fe element) was set to 0.07%, 0.15%, 0.36%, 0.58%, 0.72%, 1%, 2% and 5%, respectively [31]. The results showed that the Pb^2+^, Cd^2+^ and Cu^2+^ adsorption capacities were highest when the Fe content was set to 2%, thus magnetic sludge-based biochar with a Fe content of 2% (MSBC-2) was used in this study.

### 2.3. Characterization of Magnetic Sludge-Based Biochar

The scanning electron microscope (SEM) was used to observe the microscopic morphology (Gemini 300, Zeiss, Birmingham, UK). The X-ray photoelectron spectrometer (XPS) was used to characterize the elemental composition and chemical state of the sample surface before and after adsorption (ESCALAB 250Xi, Thermo Fisher, Waltham, MA, USA). The vibrating Sample Magnetometer (VSM) was used to determine saturation magnetization (LakeShore 7404, Westerville, OH, USA). Raman spectroscopy was measured by the Micro-Raman System 2000 (Renishaw, Wotton-under-Edge, UK). The Fourier transform infrared spectrometer (FTIR) was applied to characterize the functional groups (PerkinElmer Frontier, Waltham, MA, USA).

### 2.4. Batch Adsorption Experiments

The effects of pH (2, 3, 4, 5, and 6), temperature (25, 35 and 45 °C), ionic strength (0.005, 0.01, 0.05, 0.1 and 0.5 mol·L^−1^) and adsorbent dosage (0.1, 0.5, 1, 2 and 4 g·L^−1^) on the metal removal efficiency were determined by batch adsorption experiments. Typically, a certain amount of MSBC-2 was added to the metal ion-containing solution (Pb^2+^, Cd^2+^ and Cu^2+^) of a certain concentration and shaken orbitally at 250 rpm for 24 h at a certain temperature in the incubator. The initial solution pH was adjusted by adding either NaOH or HNO_3_. After adsorption, the mixtures were separated by centrifugation at 4000 rpm for 20 min. Then the supernatant was filtered by using a 0.45 μM PES syringe filter and diluted in 5% HNO_3_. The metal concentration was measured by inductively coupled plasma-optical emission spectroscopy (ICP-OES, Prodigy7, LeemanLabs, Hudson, NH, USA). All the experiments were conducted in triplicate.

The adsorption capacity (*Q*_e_) and removal efficiency (*E*) of metal ions onto MSBC-2 were calculated according to Equations (1) and (2).

(1) $$Q_{e}=(C_{0}-C_{e})\times \frac{V_{0}}{m}$$

(2) $$E=\frac{C_{0}-C_{e}}{C_{0}}\times 100\%$$

where *Q*_e_ is the equilibrium adsorption capacity of adsorbent for heavy metal ions, mg·g^−1^; *C*_0_ and *C*_e_ are the initial and equilibrium concentrations of heavy metal ions, mg·g^−1^; *V*_0_ is the volume of the reaction solution, L; *m* is the addition dosage of adsorbent, g; and *E* is the removal efficiency of heavy metal ions, %.

As for the kinetics experiments, samples were taken at 1, 3, 5, 10, 20, 30, 60, 90, 120, 180, 240, 360, 720, and 1440 min. Other procedures were the same as above. The results were fitted to pseudo-first-order (Equation (3)), pseudo-second-order (Equation (4)), Elovich (Equation (5)) and intraparticle diffusion (Equation (6)) models.

(3) $$\ln\;(Q_{e}-Q_{t})=\ln\;(Q_{e})-k_{1}t$$

(4) $$\frac{t}{Q_{t}}=\frac{1}{k_{2}Q_{e}{}^{2}}+\frac{t}{Q_{e}}$$

(5) $$Q_{t}=\frac{1}{b}\ln(ab)+\frac{1}{b}\ln(t)$$

(6) $$Q_{t}=K_{id}t^{0.5}+C$$

where *Q*_t_ and *Q*_e_ are the metal concentrations adsorbed at equilibrium and time *t*, mg·g^−1^; *k*_1_ is the rate constant of pseudo-first-order model, min^−1^; *k*_2_ is the rate constant of pseudo-second-order, g·(mg·min)^−1^; *a* and *b* represent the initial sorption rate constants (mg·(g·min)^−1^) and desorption constant (g·mg^-1^), respectively; *K*_id_ is the intraparticle diffusion rate constant, mg/(g·min^0.5^); and *C* reflects the boundary layer effect, mg·g^−1^.

As for the adsorption isotherm, experimental data were fitted to Langmuir (Equation (7)) and Freundlich (Equation (8)) models.

(7) $$\frac{C_{e}}{Q_{e}}=\frac{1}{Q_{m}K_{L}}+\frac{C_{e\;}}{Q_{m}}$$

(8) $$\ln(Q_{e})=\ln(K_{F})+\frac{1}{n}\ln(C_{e})$$

(9) $$R_{L}=\frac{1}{1+K_{L}C_{0}}$$

where *Q*_m_ is the maximum adsorption capacity, mg·g^−1^; *K*_L_ is the Langmuir adsorption constant, L·mg^−1^; *K*_F_ is the Freundlich constant, mg·g^−1^·(L·mg^−1^)^1/n^; *n* is the empirical heterogeneity factor; and *R*_L_ is the separation factor.

Based on *K*_L_ obtained from the Langmuir model, thermodynamic parameters were calculated by Equations (10)–(12).

(10) $$\Delta G^{0}=-RT\ln K_{L}$$

(11) $$\Delta G^{0}=\Delta H^{0}-T\Delta S^{0}$$

(12) $$\ln K_{L}=\frac{\Delta S^{0}}{R}-\frac{\Delta H^{0}}{RT}$$

where Δ*G*^0^ is the free energy change, kJ·mol^−1^; Δ*S*^0^ is the entropy change, kJ·(mol·K)^−1^; Δ*H*^0^ is the enthalpy change, kJ·mol^−1^; *R* is the ideal gas constant, 8.314 J·(mol·K)^−1^; and *T* is the thermodynamic temperature, K.

### 2.5. Statistical Analysis

The adsorption capacity (*Q*_e_) and removal efficiency (*E*) data were analyzed by using a one-way ANOVA with a significance set at *p* < 0.05 (SPSS 26.0, IBM, Armonk, NY, USA).

## 3. Results and Discussion

### 3.1. Characterization of MSBC-2

**Figure 1:**
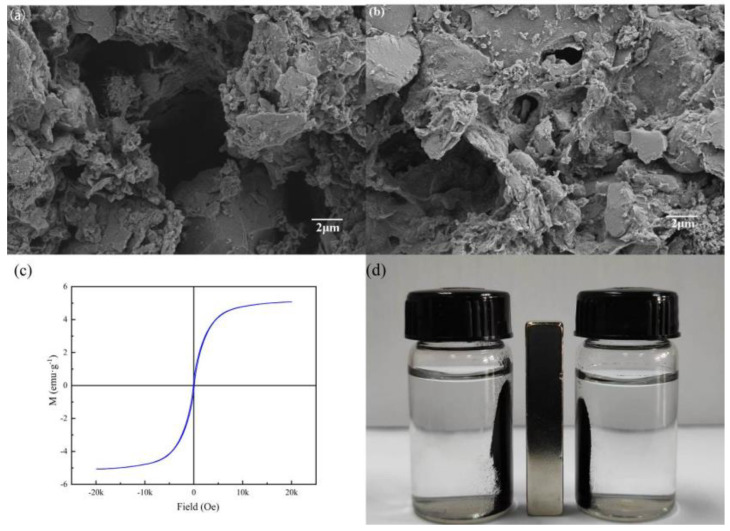
SEM surface morphology of SBC (**a**), ×5000 and MSBC-2 (**b**), ×5000, magnetization curve of MSBC-2 (**c**) and actual separation performance (**d**).

According to Figure 1a,b, the morphology of SBC was smoother, less porous and cleaner than that of MSBC-2. After magnetic modification, various nano-sized particles were exposed on the surface of the carbon skeleton. The specific surface area and pore structure of MSBC-2 and unmodified sewage sludge-derived biochar (SBC) are shown in Table 1. The specific surface area of MSBC-2 was 63.68 m^2^·g^−1^, which was higher than that of SBC (59.38 m^2^·g^−1^), due to the larger specific surface area of loaded Fe_3_O_4_ nanoparticles and the pore expansion effect brought by magnetic modification. The total pore volume of MSBC-2 (0.089 cm^3^·g^−1^) increased slightly compared to SBC (0.073 cm^3^·g^−1^). An increase in pore volume would accelerate the adsorbate entering the inner pore system of the adsorbent. The average pore diameter of MSBC-2 was 5.96 nm, implying that MSBC-2 had a typical mesoporous structure. Thus, MSBC-2 had a higher specific surface area and more abundant adsorption sites for binding metal ions.

Figure 1c indicates that the saturation magnetization value of MSBC-2 was 5.07 emu·g^−1^ and MSBC-2 had a typical superparamagnetic behaviour and high saturation magnetization value. Hence, MSBC-2 could be easily recycled from solutions by using an external magnetic field (Figure 1d).

**Figure 2:**
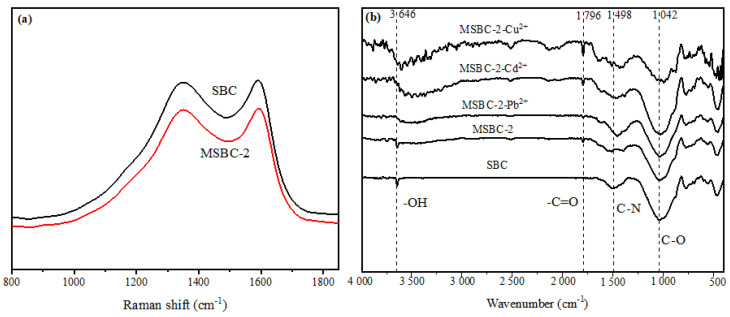
Raman spectrum (**a**) and FTIR spectrum (**b**) of SBC and MSBC-2.

Raman spectroscopy was used to explore the carbon structure (Figure 2a). As-prepared samples exhibited two peaks at approximately 1360 and 1590 cm^−1^, corresponding to the D band (disordered band) and G band (graphite band). The D-band/G-band (*I*_D_/*I*_G_) value of SBC was 1.45, while the value of MSBC-2 decreased to 1.42, indicating that magnetic modification would reduce the graphitization degree of biochar. The FTIR spectra of SBC and MSBC-2 before and after adsorption are shown in Figure 2b. The band at 3646 cm^−1^ was ascribed to the hydroxyl stretching vibration peak, and the peak intensity of the MSBC-2 was stronger than that of SBC, implying that the magnetic modification had loaded more hydroxyl groups onto MSBC-2 [32]. The appearance of a peak around 1796 cm^−1^ was observed after loading Fe, which corresponded to the stretching vibration of the carboxyl or lactone group. The result indicates that the modification process introduced more aromatic groups in biochar, which might enhance the metal adsorption capacity [33]. The band at 1498 cm^−1^ was attributed to the deformation vibration of the C-N [34].

### 3.2. The Influence of Environmental Factors

**Figure 3:**
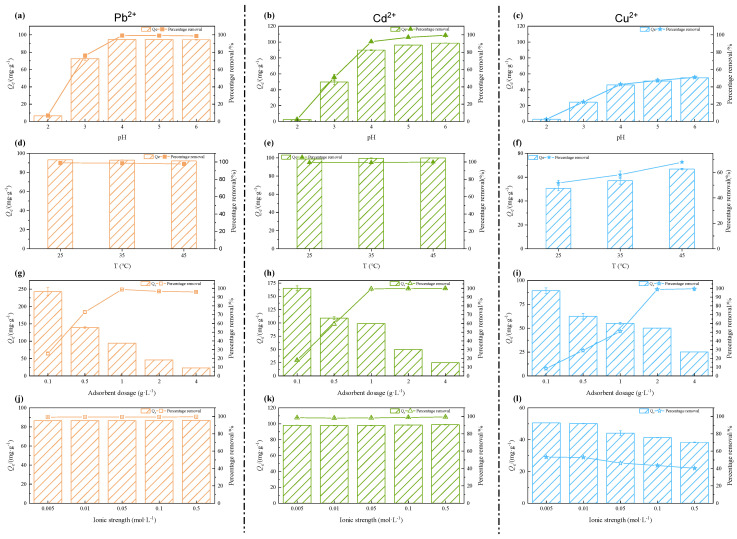
Effect of pH (**a**–**c**), temperature (**d**–**f**), adsorbent dosage (**g**–**i**) and ionic strength (**j**–**l**) on removal efficiency of Pb^2+^, Cd^2+^ and Cu^2+^ by MSBC-2.

The impacts of pH, temperature, adsorbent addition dose and ionic strength on metal removal efficiency and adsorption capacity were determined (Figure 3).

#### 3.2.1. pH

The pH value has a critical effect on the surface charge of biochar, thus significantly affecting the adsorption performance. As a higher pH would lead to precipitation, the pH range in this study was set to 2–6 for Cd^2+^, Pb^2+^ and Cu^2+^ (Figure 1a–c) [35]. For Pb^2+^ and Cd^2+^, the adsorption efficiency and capacity increased rapidly from pH 2 to 4, and then remained stable from pH 4 to 6. The maximum removal efficiencies were 98.9% for Pb^2+^ and 99.5% for Cd^2+^, respectively. For Cu^2+^, the removal efficiency did not change apparently from pH 4 to 6, which increased from 42.9% to 51.1%. The poor adsorption performance of all three metals at the pH of 2–3 was due to the higher concentration of H^+^ which would compete and occupy binding sites with heavy metal ions [36]. In addition, several functional groups such as carboxylic groups in MSBC-2 were protonated, resulting in a positive charge on the MSBC-2 and electrostatic repulsion between the heavy metal ions and MSBC-2 [37].

As the pH increased, the deprotonation process occurred and negatively charged carboxyl groups and free hydroxyl groups on the surface would provide more adsorption sites to enhance heavy metal removal [38]. The initial pH was set to 6 for the following batch adsorption experiments.

#### 3.2.2. Temperature

Figure 3e,f presents the effect of temperature on the adsorption of Pb^2+^, Cd^2+^, and Cu^2+^ by MSBC-2. At an initial heavy metal concentration of 100 mg·L^−1^, the removal efficiency of Pb^2+^ and Cd^2+^ by MSBC-2 was almost not affected by temperature due to the excessive adsorption sites, while the copper removal efficiency increased as temperature increased. When the initial Pb^2+^/Cd^2+^ concentration increased to 250 mg·L^−1^, the removal efficiency increased by 14.5% for Pb^2+^ and 18.3% for Cd^2+^, respectively, as the temperature increased from 25 °C to 45 °C. The varied influence of temperatures might be due to the different affinities of Pb^2+^, Cd^2+^, and Cu^2+^ to MBSC-2 [39,40].

#### 3.2.3. Adsorbent Dosage

The dosage of adsorbent is another important factor affecting removal efficiency. Insufficient amounts cannot achieve the purpose of treating water pollution caused by heavy metals, while excessive amounts do not make full use of resources and increase costs. The removal efficiency of Pb^2+^ and Cd^2+^ increased sharply when the MSBC-2 addition dose was increased from 0.1 g·L^−1^ to 1 g·L^−1^, then remained stable, while the inflection point occurred at 2 g·L^−1^ for Cu^2+^ (Figure 3g–i). For all three metal ions, adsorption capacities decreased as the adsorbent dosage increased. With the increasing MSBC-2 addition dose, more available adsorption sites were provided, which led to higher metal removal efficiency but reduced the adsorption per unit mass of adsorbent as well [41].

#### 3.2.4. Ionic Strength

The background ionic strengths were set to 0.005–0.5 mol·L^−1^ NaNO_3_ (Figure 3i–l). The ionic strength had a negligible effect on the removal of Pb^2+^ and Cd^2+^, suggesting that the inner complex might be generated [42]. However, the removal efficiency and adsorption capacity of Cu^2+^ by MSBC-2 decreased significantly with the increasing ionic strengths (*p* < 0.05), probably due to competition between Na^+^ and Cu^2+^ for adsorption sites [43].

### 3.3. Adsorption Kinetics

**Figure 4:**
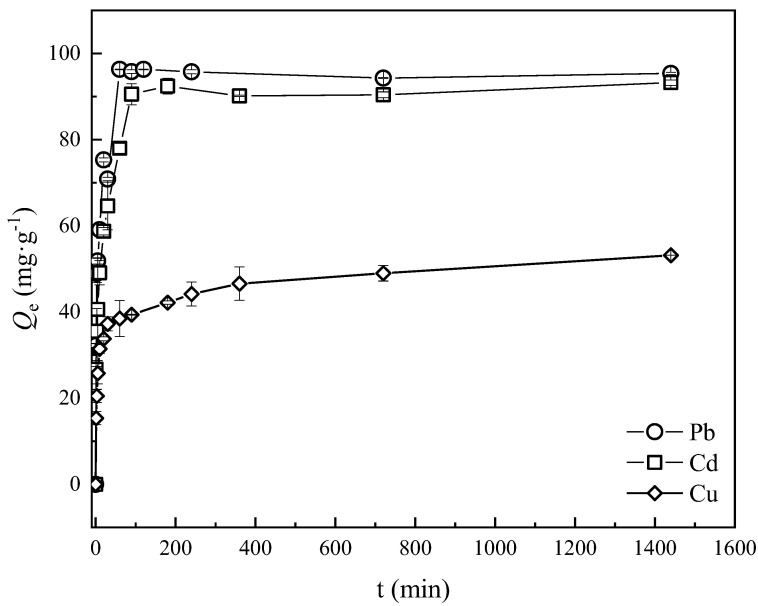
Adsorption kinetics curve of Cd^2+^, Pb^2+^ and Cu^2+^ onto MSBC-2 and SBC.

The adsorption capacity of Pb^2+^, Cd^2+^ and Cu^2+^ at different contact time is shown in Figure 4. The adsorption kinetics curves of Pb^2+^, Cd^2+^ and Cu^2+^ onto MSBC-2 were similar and the adsorption process could be divided into three stages: fast adsorption stage, slow adsorption stage and adsorption equilibrium stage. In the fast adsorption stage, the adsorption capacity and metal removal efficiency of MSBC-2 for all three metals increased rapidly, due to abundant adsorption sites on the surface of MSBC-2. Gradually, MSBC-2 adsorption sites were occupied, and then the adsorption capacity increased slowly and finally reached equilibrium. The equilibrium time was 120 min for Pb^2+^ and Cd^2+^ adsorption systems, while the Cu^2+^ adsorption onto MSBC-2 reached equilibrium at about 360 min.

To further explore the adsorption process of Pb^2+^, Cd^2+^ and Cu^2+^ onto MSBC-2, the experimental data were fitted to pseudo-first-order, pseudo-second-order, Elovich and intraparticle diffusion models (Table 2).

As for Pb^2+^, Cd^2+^ and Cu^2+^, the correlation coefficients (*R*^2^) of the intraparticle model (*R*^2^ = 0.4–0.74) were lower than the fits to the other three models (*R*^2^ = 0.74–1.00), indicating that intraparticle diffusion was not the only rate-limiting step during the adsorption process [44]. All kinetics data could be described best by the pseudo-second-order model with the *R*^2^ over 0.99, indicating that chemisorption was the most responsible rate-limiting step for adsorption of Pb^2+^, Cd^2+^ and Cu^2+^ onto MSBC-2 [45,46]. Moreover, the adsorption rates for the three metals were various, and the rate constant sequence was: Pb^2+^ > Cd^2+^ > Cu^2+^. The Elovich model generally presents the heterogeneous chemisorption process [44]. The experimental data for the Elovich models gave high correlation coefficients for Cd^2+^ (*R*^2^ = 0.9124, *p* < 0.0001) and Cu^2+^ (*R*^2^ = 0.9794, *p* < 0.0001), implying the adsorption systems were highly heterogeneous.

### 3.4. Adsorption Isotherms

**Figure 5:**
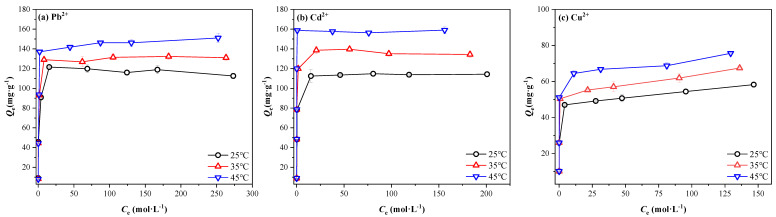
The adsorption curves of Cd^2+^, Pb^2+^ and Cu^2+^ onto MSCB-2 at different temperatures.

Figure 5 shows the adsorption isotherms of Pb^2+^, Cd^2+^ and Cu^2+^ onto MSBC-2 at 25 °C, 35 °C and 45 °C. As the initial metal concentrations increased, the adsorption capacities of Pb^2+^, Cd^2+^ and Cu^2+^ by MSBC-2 increased gradually and then tended to reach equilibrium. The adsorption capacities of the three metals were greater at a higher temperature. To further illustrate the adsorption mechanism of Pb^2+^, Cd^2+^ and Cu^2+^ onto MSBC-2, Langmuir and Freundlich models were used to fit the experimental data (Table 3). Compared to Freundlich model, the Langmuir model showed better fits to the experimental data with *R*^2^ of 0.9958–0.9999. The results revealed that adsorption processes were monolayer adsorption between the metal ions and oxygen-containing functional groups distributed homogeneously on the MSBC-2 surface [47,48]. When the reaction temperature increased from 25 °C to 45 °C, the maximum adsorption capacities increased from 113.64 mg·g^−1^ to 151.52 mg·g^−1^ for Pb^2+^, from 101.01 mg·g^−1^ to 109.89 mg·g^−1^ for Cd^2+^ and from 57.80 mg·g^−1^ to 74.07 mg·g^−1^ for Cu^2+^, respectively. The adsorption processes were endothermic for all three metal ions.

The separation factor *R*_L_ (Equation (9)) was calculated from the Langmuir model, which can indicate whether adsorption is favourable (0 < *R*_L_ < 1), linear (*R*_L_ = 1), unfavourable (*R*_L_ > 1) or irreversible (*R*_L_ = 0) [49]. As shown in Table 3, *R*_L_ values ranged from 0.0011 to 0.2278, thus the adsorption of Pb^2+^, Cd^2+^ and Cu^2+^ onto MSBC-2 at various temperatures was favourable. The 1/*n* value represents the degree of heterogeneity [50]. The 1/*n* values were all less than 1, proving that the adsorption of Pb^2+^, Cd^2+^ and Cu^2+^ onto MSBC-2 was favourable, which was in accordance with *R*_L_ values [51].

### 3.5. Adsorption Thermodynamic Analysis

Based on the *K*_L_ values achieved from Langmuir model, the thermodynamic parameters were calculated and presented in Table 4. Negative Δ*G*^0^ values implied the adsorption of Pb^2+^, Cd^2+^ and Cu^2+^ onto MSBC-2 was spontaneous [52]. With the increasing temperatures, Δ*G*^0^ decreased for all three metals illustrating that the adsorption process was more spontaneous and favourable at higher temperatures [53]. This could be due to the enhancement of the adsorbate molecules mobility, indicating greater affinity at the higher temperature. The sorting of Δ*G*^0^ values was: Cu^2+^ > Pb^2+^ > Cd^2+^, implying that the adsorption of Cd^2+^ onto MSBC-2 had the highest spontaneity and largest feasibility. All the Δ*H*^0^ values were positive, indicating that the adsorption of Pb^2+^, Cd^2+^ and Cu^2+^ onto MSBC-2 was endothermic in nature, which was consistent with the adsorption isotherms results. Typically, the value of Δ*H*^0^ in a range of 80–200 kJ·mol^−1^ suggests chemisorption [54,55]. The calculated Δ*H*^0^ values were 91.41 kJ·mol^−1^, 186.89 kJ·mol^−1^ and 46.28 kJ·mol^−1^ for Pb^2+^, Cd^2+^ and Cu^2+^, respectively. The results imply that the adsorption of the three metal ions by MSBC-2 mainly depended on chemical adsorption, which agreed with the adsorption kinetics and adsorption isotherm results. The positive Δ*S* values exhibited that the randomness on the metal solution-MSBC interface increased and the affinity of metal ions was adequate to adhere to the adsorbent surface during the sorption process [56].

### 3.6. Adsorption Mechanisms

To further explore the adsorption mechanism, FTIR and XPS analyses were performed by identifying the changes in functional groups before and after adsorption. According to FTIR, the peak intensity of the –OH group was weakened after the adsorption of heavy metals, probably due to the involvement of the –OH group in surface complexation and ion exchange. The intensity of the peak at 1042 cm^−1^, which was ascribed to C–O stretching vibration, reduced after metal adsorption, suggesting that C–O was involved in the formation of chelates with metals [57].

**Figure 6:**
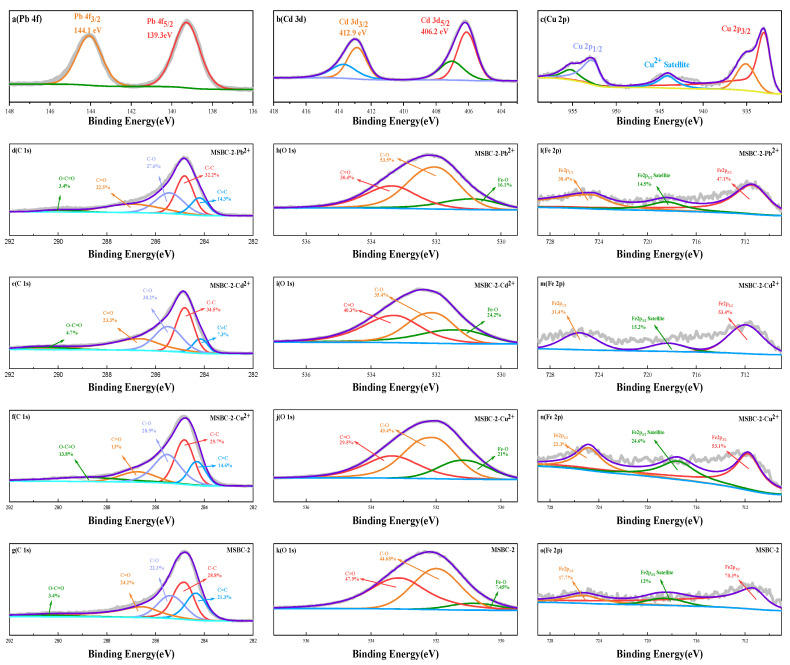
XPS analysis of MSBC-2 before and after Cd^2+^, Pb^2+^ and Cu^2+^ adsorption.

The XPS spectroscopy was used to investigate the elemental composition and chemical state of MSBC-2 before and after adsorption (Figure 6). The Fe 2p spectrum show two peaks representing Fe 2p_3/2_ (711.3 eV) and Fe 2p_1/2_ (725.3 eV), proving that iron was loaded onto biochar successfully [58]. The characteristic binding energies ascribed to Pb 4f (139.3 and 14.1 eV), Cd 3d (412.9 and 406.2 eV) and Cu 2p (411.9 and 405.1 eV) occurred after adsorption, indicating that all three metal ions were adsorbed by MSBC-2 [59]. Specifically, the peaks with the binding energies of 139.3 and 144.1 eV in the Pb 4f were attributed to Pb^2+^ and Pb-O, and the existence of Pb^2+^ species exhibited that electrostatic attraction occurred between Pb and MSBC-2 [60]. The Cd 3d could be divided into Cd-π (412.9 eV) and Cd-O binding (406.2 eV), suggesting that coordination with π electrons and electrostatic attraction were involved in the Cd^2+^ adsorption process [61,62]. The spectra of Cu 2p could be divided into three main peaks Cu 2p1/2, Cu 2p3/2, and shake-up satellites. The peaks with the binding energy of 934.0 eV in the Cu 2d were ascribed to (–COO)_2_Cu and (–O)_2_Cu, representing that the carboxyl and hydroxyl groups reacted with the Cu^2+^ [63]. The appearance of the shake-up satellites demonstrates that an ion exchange existed in the Cu^2+^ adsorption process [64]. The O 1s XPS spectra of the MSBC-2 was decomposed into three components: Fe–O–H, Fe–O–Pb, Fe–O–Cd, or Fe–O–Cu at ~531.5 eV, C–O at 532.4 eV and O=C–O at 533.6 eV, respectively. After metal ions were adsorbed by MSBC-2, the intensity of the peaks at ~531.5 eV increased from 7.5% to 16.1% for the Pb^2+^ system, to 24.2% for the Cd^2+^ system and to 21.0% for the Cu^2+^ system, due to the interaction of the surface of Fe–O–H with Pb^2+^/Cd^2+^/Cu^2+^ through ligand exchange (O-Pb, O-Cd or O-Cd) [62,65]. The O=C-O peak decreased from 47.9% to 30.4% (for Pb^2+^), 35.4% (for Cd^2+^) and 29.5% (for Cu^2+^), respectively, implying that −COOH groups were involved in surface complexation [65].

In conclusion, surface complexation, cation-π interaction, ion exchange and electrostatic attraction were involved in the adsorption of Pb^2+^, Cd^2+^ and Cu^2+^ onto MSBC-2.

### 3.7. Comparison with Other Relevant Adsorbents

Table 5 summarizes several adsorption parameters (e.g., *Q*_m_ and *k*_2_) of as-prepared material and other relevant adsorbents. The maximum adsorption capacities of MSBC-2 for Cd^2+^, Pb^2+^ and Cu^2+^ were greater than those of most resembling adsorbents, thus MSBC-2 could be used as a potential adsorbent for Pb^2+^, Cd^2+^ and Cd^2+^ removal.

## 4. Conclusions

To address the drawback of two-step and one-pot synthesis methods for producing biochar, a magnetic sewage sludge-derived biochar (MSBC-2) was synthesized by a solvent-free method in this study. The adsorption performance of three metal ions (Pb^2+^, Cd^2+^ and Cu^2+^) onto the MSBC-2 was investigated in detail together with adsorption mechanisms. The pseudo-second-order kinetic model (*R*^2^ = 0.9971–0.9999, *p*< 0.0001) and Langmuir model (*R*^2^ = 0.9958–0.9999, *p* < 0.0001) provided the best fit to the experimental data, implying a monolayered chemisorption process of Pb^2+^, Cd^2+^and Cu^2+^ onto MSBC-2. As the temperature increased from 25 °C to 45 °C, the maximum adsorption capacities increased from 113.64 mg·g^−1^ to 151.52 mg·g^−1^ for Pb^2+^, from 101.01 mg·g^−1^ to 109.89 mg·g^−1^ for Cd^2+^ and from 57.80 mg·g^−1^ to 74.07 mg·g^−1^ for Cu^2+^, respectively. Thermodynamic parameters (Δ*G*^0^ < 0, Δ*S*^0^ > 0, Δ*H*^0^ > 0) demonstrated that the adsorption processes of all three metals by MSBC-2 were favourable, spontaneous and endothermic. The adsorption mechanisms involved surface complexation, cation-π interaction, ion exchange and electrostatic attraction for the adsorption of Pb^2+^, Cd^2+^ and Cu^2+^ onto MSBC-2. Overall, this study will provide a new perspective for the synthesis of magnetic biochar and MSBC-2 presents a significant potential as an adsorbent for heavy metal removal.

## Figures and Tables

**Table 1:** Specific surface area and pore structure characteristics of MSBC-2 and SBC [31].

Sample	BET (m^2^·g^−1^)	Total Pore Volume (cm^3^·g^−1^)	Average Pore Diameter (nm)
MSBC-2	63.68	0.089	5.96
SBC	59.38	0.073	4.56

**Table 2:** Adsorption kinetics parameters for various adsorption kinetics models.

Metal	*Q*_e,exp_ (mg·g^−1^)	Pseudo-First-Order Model				Pseudo-Second-Order Model				Elovich Model				Intraparticle Diffusion Model			
		*Q*_e,cal_ (mg·g^−1^)	*k*_1_(min^−1^)	*R* ^2^	*p*	*Q*_e,cal_ (mg·g^−1^)	*k*_2_ (g·(mg·min)^−1^)	*R* ^2^	*p*	*a* (mg·(g·min)^−1^)	*b* (g·mg^−1^)	*R* ^2^	*p*	*K*_id_ (mg·(g·min^0.5^)^−1^)	*C* (mg·g^−1^)	*R* ^2^	*p*
Pb	95.75	44.35	4.80 × 10^−3^	0.8788	0.0006	95.24	3.33 × 10^−3^	0.9999	<0.0001	6.75 × 10^2^	0.104	0.8392	<0.0001	1.333	61.94	0.4263	0.0213
Cd	93.22	44.33	4.60 × 10^−3^	0.7896	0.0006	93.46	1.27 × 10^−3^	0.9998	<0.0001	1.53 × 10^2^	0.095	0.9124	<0.0001	1.638	49.41	0.5750	0.0043
Cu	53.17	24.39	3.00 × 10^−3^	0.7398	0.0002	52.91	8.60 × 10^−3^	0.9971	<0.0001	6.75 × 10^2^	0.201	0.9794	<0.0001	0.862	26.87	0.7350	0.0002

**Table 3:** Adsorption isotherm parameters of heavy metals onto MSBC-2 at different temperatures for Langmuir and Freundlich models.

Metal	Temperature (°C)	Langmuir					Freundlich			
		*Q*_m_ (mg·g^−1^)	*K*_L_ (L·mg^−1^)	*R* ^2^	*p*	*R*_L_	*K*_f_ (mg·g^−1^·(L·mg^−1^)^1/n^)	*n*	*R* ^2^	*p*
Pb	25	113.64	1.073	0.9990	<0.0001	0.0011–0.0458	39.958	4.024	0.7527	0.0052
	35	131.58	1.310	0.9999	<0.0001	0.0020–0.0768	44.228	3.986	0.6709	0.0129
	45	151.52	0.617	0.9997	<0.0001	0.0042–0.1501	49.511	3.849	0.6036	0.0233
Cd	25	101.01	0.339	0.9992	<0.0001	0.0073–0.2278	34.03	4.40	0.5379	0.0384
	35	106.38	0.355	0.9997	<0.0001	0.0070–0.2198	33.08	4.06	0.5890	0.0262
	45	109.89	0.387	0.9996	<0.0001	0.0064–0.2053	35.08	4.13	0.5601	0.0327
Cu	25	57.80	0.413	0.9968	<0.0001	0.0060–0.1949	27.183	5.89	0.7695	0.0095
	35	66.23	0.557	0.9958	<0.0001	0.0045–0.1522	32.122	6.03	0.7258	0.0149
	45	74.07	0.808	0.9966	<0.0001	0.0031–0.1101	41.04	6.89	0.7231	0.0153

**Table 4:** Thermodynamic parameters of Cd^2+^, Pb^2+^ and Cu^2+^ onto MSBC-2.

Metal	*T* (K)	Δ*G*^0^ (kJ·mol^−1^)	Δ*S*^0^ (kJ·(mol·K)^−1^)	Δ*H*^0^ (kJ·mol^−1^)	*R* ^2^	*p*
Pb	298.15	−4.8321	0.3218	91.4139	0.9631	<0.05
	308.15	−7.0649				
	318.15	−11.3108				
Cd	298.15	−4.9783	0.6430	186.8869	0.9973	<0.05
	308.15	−10.8810				
	318.15	−17.8607				
Cu	298.15	−1.4316	0.1594	46.2807	0.9464	<0.05
	308.15	−2.4190				
	318.15	−4.6460				

**Table 5:** Comparison of *Q*_m_ and *k*_2_ between MSBC-2 and various magnetic biochars.

Raw Materials	*k*_2_ (g·(mg·min)^−1^)	*Q*_m_ (mg·g^−1^)	Reference
Rice straw	2.4 × 10^−2^ (Pb) 9.00 × 10^−2^ (Cd) 3.90 × 10^−2^ (Cu)	133.3 (Pb) 42.7 (Cd) 19.6 (Cu)	[66]
Date leaves and stalks	3.98 × 10^−3^ (Pb) 2.70 × 10^−3^ (Cd)	103.1 (Pb) 106.4 (Cd)	[18]
Cellulose	5.00 × 10^−3^ (Pb) 5.00 × 10^−4^ (Cu)	17.3 (Pb) 42.2 (Cu)	[67]
Corn straw	3.2 × 10^−4^ (Pb) 1.30 × 10^−4^ (Cd) 6.10 × 10^−4^ (Cu)	54.5 (Pb) 66.2 (Cd) 84.8 (Cu)	[68]
Rice husk	5.00 × 10^−2^ (Cd)	21.7 (Cd)	[69]
Sunflower	2.8 × 10^−2^ (Cd) 2.3 × 10^−2^ (Cu)	2.9 (Cd) 2.7 (Cu)	[70]
Chitosan	4.5 × 10^−3^ (Cu)	33.9 (Cu)	[71]
Cane	6.33 × 10^−4^ (Pb)	40.6 (Pb)	[72]
Sludge	3.33 × 10^−3^ (Pb) 1.27 × 10^−3^ (Cd) 8.60 × 10^−4^ (Cu)	113.6 (Pb) 101.0 (Cd) 57.8 (Cu)	This study

## Data Availability

The data presented in this study are available on request from the corresponding author.
